# Supplementary material for: The effects of multi-colour light filtering glasses on human brain wave activity
Source: BMC Neurosci. 2024 Apr 12;25:21. doi: 10.1186/s12868-024-00865-0 (PMC11015570; doi:10.1186/s12868-024-00865-0)
Supplement: Supplementary file 1 — Supplementary Material 1 [file 12868_2024_865_MOESM1_ESM.pdf]

Lens Test Report

Measured by Topcon TM-3

样品	TD011 POLARIZED RED LENS	检验员	eric
公司	Ningbo Ning Shing Industrial Co., Ltd.	电话	0086 577 8844 8899
地址	No.77, Haoxin Road, Shanghui, Ouhai district, Wenzhou 325000, China		
电邮		传真	0086 577 8606 6383

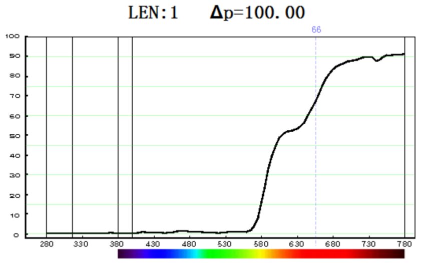

Standard: BS EN ISO 12312-1:2013+A1:2015

Item	Value	Requirement	Result
Luminous Transmittance(Tv)	13.37%		
Filter Category	3		
Tsuvb (280nm-315nm)	0.01%	%(1.0)	PASS
Tsuva (315nm-380nm)	0.04%	<6.69%(0.5Tv)	PASS
Tmin (475nm-650nm)	0.30%	>=2.67%(0.2Tv)	REF.
************************ Claimed Value ************************			
Tsuvb (280nm-315nm)	0.01%		
Tsuva (315nm-380nm)	0.04%		
Tsuv (280nm-380nm)	0.03%		
Tsb (380nm-500nm)	0.73%	<=(95.00+0.5)%	PASS
***************** Recognition of signal lights *****************			
Red [Q] (INCT)	3.92	>=0.8	PASS
Yellow [Q] (INCT)	1.91	>=0.6	PASS
Green [Q] (INCT)	0.28	>=0.6	REF.
Blue [Q] (INCT)	0.35	>=0.6	REF.

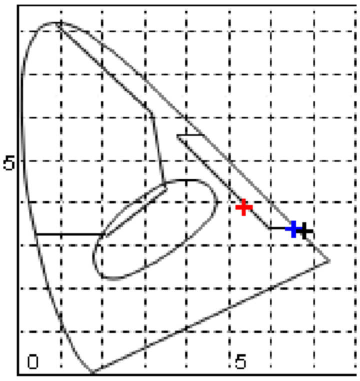

Standard: ANSI Z80.3-2015

Item	Value	Requirement	Result
High and prolonged exposure	yes		
Luminous Transmittance(Tc)	13.60%		
Filter Category	(General Purpose, medium to dark)		
************************* Tsig Vaules *************************			
RED	59.07%	>=8%	PASS
YELLOW	27.46%	>=6%	PASS
GREEN	3.62%	>=6%	REF.
********************** Mean transmittance **********************			
Tsb (380nm-500nm)	0.73%		
UVB (280nm-315nm)	0.01%	<=1%	PASS
UVA (315nm-380nm)	0.06%	<=6.80%(0.5Tv)	PASS
Tmin (475nm-650nm)	0.30%	>=2.72%(0.2Tv)	REF.
**************************** Color ****************************			
DAY LIGHT	X:0.635 Y:0.344		REF.
YELLOW	X:0.663 Y:0.336		REF.
GREEN	X:0.522 Y:0.392		REF.

WL	T	WL	T	WL	T	WL	T
280	0.02	410	0.58	540	0.86	670	78.63
285	0.01	415	0.72	545	0.87	675	81.17
290	0.02	420	0.66	550	0.80	680	83.31
295	0.02	425	0.63	555	0.78	685	84.82
300	0.02	430	0.64	560	1.03	690	85.89
305	0.02	435	0.55	565	1.79	695	86.69
310	0.00	440	0.37	570	3.73	700	87.26
315	0.00	445	0.28	575	7.84	705	87.87
320	0.00	450	0.32	580	15.03	710	88.28
325	0.00	455	0.58	585	24.00	715	88.70
330	0.00	460	1.06	590	32.69	720	89.15
335	0.00	465	1.40	595	39.64	725	89.52
340	0.00	470	1.39	600	45.05	730	89.87
345	0.00	475	1.21	605	48.52	735	89.72
350	0.00	480	1.01	610	50.52	740	87.88
355	0.00	485	0.86	615	51.65	745	88.28
360	0.00	490	0.78	620	52.14	750	89.75
365	0.02	495	0.71	625	52.64	755	90.36
370	0.32	500	0.63	630	53.33	760	90.69
375	0.27	505	0.50	635	54.60	765	90.88
380	0.21	510	0.38	640	56.58	770	90.81
385	0.13	515	0.31	645	59.53	775	90.92
390	0.11	520	0.30	650	63.26	780	91.33
395	0.09	525	0.49	655	66.85		
400	0.09	530	0.61	660	70.35		
405	0.23	535	0.76	665	75.32		

Standard: AS/NZS 1067.1:2016

Item	Value	Requirement	Result
Luminous Transmittance(Tv)	13.37%		
Filter Category	3		
Tsuvb (280nm-315nm)	0.01%	%(1.0)	PASS
Tsuva (315nm-400nm)	0.04%	<6.69%(0.5Tv)	PASS
Tmin (475nm-650nm)	0.30%	>=2.67%(0.2Tv)	REF.
************************ Claimed Value ************************			
Tsuvb (280nm-315nm)	0.01%	<=(10.00+0.5)%	PASS
Tsuva (315nm-400nm)	0.04%	<=(10.00+0.5)%	PASS
Tsuv (280nm-400nm)	0.03%	<=(10.00+0.5)%	PASS
Tsb (380nm-500nm)	0.73%	<=(95.00+0.5)%	PASS
***************** Recognition of signal lights *****************			
Red [Q] (INCT)	3.92	>=0.8	PASS
Yellow [Q] (INCT)	1.91	>=0.6	PASS
Green [Q] (INCT)	0.28	>=0.6	REF.
Blue [Q] (INCT)	0.35	>=0.7	REF.

UV400 0.03%

*** CIE 1976 L*,a*,b* color space coordinates,illuminan D65 ***

L* = 100.00 a* = 0.00 b* = 160.81
